# Parental mediation profiles across six European countries: a person-centered analysis using three-wave longitudinal data

**DOI:** 10.3389/fpsyg.2026.1858309

**Published:** 2026-06-22

**Authors:** Zhuo Wang

**Affiliations:** Department of Primary Education, Qingdao University, Qingdao, China

**Keywords:** adolescence, cross-national comparison, digital media, latent profile analysis, longitudinal stability, parental mediation, Person-centered approach, ySKILLS

## Abstract

**Background:**

Parents use diverse strategies to mediate children's digital media use, yet prior research has largely examined them in isolation using variable-centered approaches. The few person-centered studies use single-country samples and have not validated longitudinal profile stability.

**Methods:**

Latent profile analysis (LPA) and latent transition analysis (LTA) were applied to three-wave longitudinal data from the ySKILLS project (six European countries: Estonia, Finland, Germany, Italy, Poland, Portugal; ages 11–20), using Mplus 8.3 with robust maximum likelihood (MLR) and full-information maximum likelihood (FIML) for missing data. The analytic sample comprised *N* = 9,881 participants with valid data on at least one mediation indicator across waves (W1 *N* = 5,675; W2 *N* = 6,238; W3 *N* = 5,472); the cross-sectional EFA used *N* = 5,833 (W1, eight items). Two standardized composites (restrictive, enabling mediation) served as LPA indicators; a monitoring item was reserved for external validation. Cross-national distributions were tested with χ^2^ and Cramér's V; distal outcomes (six wellbeing variables) with the BCH method; stability and country moderation via three-wave and multi-group LTA (measurement invariance).

**Results:**

Three profiles emerged: Disengaged (40.2%), Moderate Balanced (35.4%), and Active All-Round (24.4%). Profile validity was confirmed against monitoring (*F*(2, 5,410) = 910.80, *p* < .001, η^2^ = 0.252). Prevalence varied significantly across countries (χ^2^ (10) = 365.63, *p* < 0.001, Cramér's *V* = 0.179): Estonia had the most Disengaged adolescents (58.4%; ASR = +13.4), Portugal the most Active All-Round (33.6%; ASR = +8.5). BCH analyses revealed a threshold rather than gradient pattern: Disengaged adolescents showed less favorable outcomes than both Moderate and Active profiles, which differed less consistently. Multi-group LTA revealed uniform drift toward Disengaged across all six countries from W1 to W3 (2-year net increases: +12.1–+25.2 pp), with Disengaged highly absorbing in every country (one-wave stability: 0.830–0.965).

**Conclusion:**

Parental mediation strategies co-occur in interpretable configurations that vary across cultures and developmental stages. Disengaged adolescents showed less favorable outcomes than those whose parents engaged at any level (threshold pattern), supporting basic engagement over its absence. The absorbing nature of disengagement across all six countries underscores the need for universal early-adolescence interventions before it consolidates.

## 1 Introduction

The proliferation of digital devices has fundamentally transformed childhood and adolescence. European youth now spend an average of 2–4 h daily online (Smahel et al., 2020), and in some countries, 96% of young people report daily internet use (Liu et al., 2024). This pervasive digital engagement presents a dual challenge for parents: it offers unprecedented opportunities for learning, social connection, and creative expression, while simultaneously exposing children to risks including cyberbullying, inappropriate content, and problematic use patterns (Livingstone et al., 2017). Two-thirds of today's parents feel that parenting is harder than it was 20 years ago, with over a quarter citing technology as the primary source of difficulty (Navarro and Jensen, 2026).

In response to these challenges, parents deploy a range of strategies—collectively termed parental mediation—to regulate, guide, and support their children's digital lives. A substantial body of research has examined the effectiveness of different mediation strategies, culminating in two major meta-analyses. Chen and Shi (2019), synthesizing 52 studies representing 74,159 participants, found that restrictive mediation was more effective than active mediation in decreasing screen time, but active mediation and co-use were superior for reducing media-related risks such as cyberbullying and sexual content exposure. More recently, Tan et al. (2025), in a comprehensive three-level meta-analysis of 88 studies spanning 2008–2024, reached a nuanced conclusion:

“*there is no one-size-fits-all approach to digital parenting for comprehensively enhancing children's digital wellbeing*.” (p. 1)

Positive mediation was most strongly linked to positive digital wellbeing, while co-use was most protective against negative digital wellbeing. These findings underscore that different strategies serve distinct functions, raising the question of how parents combine them in practice. Throughout this paper we use “parental mediation” as shorthand; because all measurements derive from adolescents' self-reports, the construct of interest is adolescents' perceptions of parental mediation, rather than directly observed parental behavior. Documented discrepancies between parent and child reports of the same mediation acts (e.g., Symons et al., 2017; Mayen and Camerini, 2025) make this distinction theoretically and methodologically consequential, and we maintain a child-perspective framing throughout.

### 1.1 Parental mediation: conceptual framework

Parental mediation theory (PMT; Valkenburg et al., 1999) distinguishes four practices through which parents manage children's media use. Restrictive mediation sets rules limiting when, how long, and what content children may access. Active (enabling) mediation involves talking with children about online content and encouraging safe, exploratory use. Co-use refers to parents sharing the media experience alongside the child, thereby modeling media behavior. Monitoring involves surveillance of children's online activity, such as checking browsing history or social-media accounts. Originally developed for television, the framework was extended to online monitoring as digital media made oversight increasingly necessary (Livingstone and Helsper, 2008).

These dimensions are theoretically distinct but empirically correlated. In the present dataset, restrictive and enabling mediation correlated at *r* = 0.40, and both correlated substantially with monitoring (*r* = 0.52 and 0.51, respectively), indicating that parents who engage in one form of mediation tend to employ others as well. Helsper et al. (2024), analyzing data from eight European countries, found that parents with higher perceived control over online risks and broader digital skills engaged in more mediation across all dimensions, suggesting a common underlying factor of “parental digital engagement.”

Research on strategy effectiveness has produced a central theoretical puzzle: the “risk-opportunity paradox” (Livingstone and Helsper, 2010). Restrictive mediation reduces risk exposure but simultaneously constrains digital opportunities and skill development (Chen and Shi, 2019; Kalmus et al., 2022). Enabling mediation promotes digital skills and online opportunities but can paradoxically increase risk exposure by encouraging broader internet use (Duerager and Livingstone, 2012). This paradox suggests that no single strategy optimally balances protection against risk with preservation of opportunities. A critical question is whether certain combinations of strategies—rather than any strategy in isolation—can resolve this tension.

### 1.2 Theoretical integration: self-determination theory and authoritative parenting

We integrate four traditions into a single deductive chain. First, PMT specifies the typology of mediation strategies as perceived by the child (H1). Second, Bronfenbrenner's (1979) ecological systems theory locates the parent–child microsystem within mesosystem (school, peer) and macrosystem (cultural norms, welfare-state regimes, digital infrastructure) layers — providing the rationale for cross-national variation (H2). Third, Self-Determination Theory (Ryan and Deci, 2000) and Baumrind's (1991) authoritative parenting framework specify the proximal mechanism by which configurations of mediation translate into child wellbeing (H3): comprehensive mediation is hypothesized to satisfy the child's needs for autonomy-supportive structure, competence, and relatedness. Fourth, Steinberg's (2001) developmental account of adolescent autonomy-seeking specifies the temporal trajectory: as adolescents mature, parental engagement may attenuate (H4). Each hypothesis below is re-derived from this integrated chain.

Self-Determination Theory (SDT; Ryan and Deci, 2000) provides a complementary lens for understanding parental mediation. SDT posits that human wellbeing depends on the satisfaction of three basic psychological needs: autonomy (experiencing volition and choice), competence (feeling effective), and relatedness (feeling connected to others). Applied to digital parenting, SDT predicts that mediation strategies that support children's autonomy (e.g., discussing reasons for rules, encouraging independent exploration) should promote healthier digital engagement than strategies that thwart autonomy (e.g., covert monitoring, unilateral restrictions without explanation). Mayen and Camerini (2025), drawing on SDT, found that Swiss adolescents in their “Moderate” mediation class reported lower perceived autonomy than those in the “Enforcing and Engaged” class—a counterintuitive finding suggesting that moderate, uncommitted mediation may be more autonomy-undermining than clear, communicated boundaries. We apply SDT exclusively at the level of the adolescent's experience: the question is whether different perceived configurations of restrictive and enabling mediation differentially satisfy the child's needs for autonomy, competence, and relatedness. Parents' own psychological needs, digital competence, and parenting stress — which are also predictive of mediation behavior (e.g., Helsper et al., 2024) — were not measured in ySKILLS and are flagged in the Limitations as a gap requiring complementary parent-report studies.

This aligns with Baumrind's (1991) authoritative parenting framework, which holds that optimal child outcomes result from the combination of high warmth (responsiveness, communication) and high structure (clear expectations, consistent rules)—not from either dimension alone. In digital parenting terms, authoritative mediation would combine enabling strategies (high warmth/communication) with restrictive strategies (high structure/rules). Navarro and Jensen (2026), applying LPA to a U.S. sample, found that their mediation profiles

“*mirrored general parenting styles to a limited degree*,” (p. 481)

with support for authoritative and uninvolved patterns but not for authoritarian (high restriction, low enabling) or indulgent (high enabling, low restriction) patterns. Whether this correspondence holds cross-nationally is an open question.

### 1.3 The need for person-centered approaches

The vast majority of parental mediation research has adopted a variable-centered approach, treating each mediation dimension as an independent predictor of child outcomes (Kalmus et al., 2022). This approach implicitly assumes that the effect of one strategy is constant regardless of concurrent strategies. In reality, however, parents do not use strategies in isolation. They combine them into configurations—a parent who actively discusses online safety may also set screen time limits and install filtering software, while another parent may rely primarily on rules with minimal communication. These naturally occurring combinations cannot be captured by examining dimensions independently.

Person-centered approaches such as latent profile analysis (LPA) address this limitation by identifying subgroups of individuals who share similar patterns across multiple dimensions simultaneously (Spurk et al., 2020). LPA is a model-based approach that provides formal statistical criteria for determining the optimal number of profiles, including the Bayesian Information Criterion (BIC), entropy, and the bootstrap likelihood ratio test (Nylund-Gibson and Choi, 2018). Unlike cluster analysis, LPA assigns probabilistic rather than deterministic class memberships, providing classification uncertainty estimates. Spurk et al. (2020) identified three key advantages over variable-centered methods: (a) LPA captures naturally occurring combinations that may not be apparent from bivariate correlations; (b) it allows identification of qualitatively distinct subgroups rather than assuming a single population; and (c) classification probabilities quantify assignment certainty. These features make LPA particularly suited for studying parental mediation, where the theoretical expectation from both PMT and authoritative parenting theory is that parents combine strategies in distinct ways with differential effects on child outcomes.

### 1.4 Person-centered studies of parental mediation

A small but rapidly growing body of research has applied LPA or related methods to parental mediation, revealing distinct profiles across diverse cultural contexts.

In the United States, Navarro and Jensen (2026) used a multidimensional mediation measure with a nationally representative sample and identified four latent profiles: high Digital Mediators, Average Digital Mediators, Moderately Involved, and Digitally Uninvolved. These profiles were generalizable across mothers and fathers and differentially associated with parent income, child age, and technology-related confidence. Importantly, parent screen time, child screen time, the presence of an adolescent in the home, and parents' technology-related confidence were positively related to membership in the more engaged profiles, suggesting that digital familiarity promotes rather than substitutes for active mediation.

In Switzerland, Mayen and Camerini (2025) applied Latent Transition Analysis (LTA) to three-wave panel data from 717 adolescents (2018–2020), identifying three classes: Enforcing and Engaged, Hands-off, and Moderate. Profiles showed fluctuation over time, with adolescents transitioning between classes across waves. A critical finding was that the Moderate class—not the Hands-off class—reported the lowest perceived autonomy, challenging the intuitive assumption that less mediation promotes greater autonomy. The authors interpreted this through an SDT lens, arguing that clear, engaged mediation (even when restrictive) provides a more autonomy-supportive context than ambiguous, inconsistent mediation.

In Spain, Sevilla-Fernández et al. (2026) analyzed data from 4,371 students across 11 regions using a six-dimensional parental mediation measure (active mediation of internet use, active mediation of internet safety, child-initiated mediation, parental monitoring, technical controls, and restrictive mediation). They identified four profiles: integral mediation (20.9%), proactive mediation (25.6%), technological mediation (26.2%), and minimal mediation (27.3%). A key finding was that.

“*the amount of mediation is more relevant than the specific strategy*” (p. 491)

The integral profile, which reflected high scores across all six dimensions, was associated with the lowest screen time and social media use. This suggests that mediation intensity may matter more than mediation type, a proposition our study can test with cross-national data.

In China, Li et al. (2024) examined parental mediation among 1,415 families with children aged 3–6 in Shanghai and identified four profiles reflecting the interplay between maternal and paternal involvement: mother-dominated mediation, father-dominated mediation, coordinated high-level mediation, and coordinated low-level mediation. Children in the coordinated high-level group showed the lowest probability of problematic media use, while those in the coordinated low-level group showed the highest. The study further revealed that parental age, education, and marital conflict predicted profile membership, indicating that mediation configurations are embedded within broader family dynamics. In a rural Chinese context, Ren et al. (2026) identified four profiles among 713 children and found that “communicative regulators” showed the highest digital resilience, reinforcing the importance of communicative elements in effective mediation.

In the family media domain more broadly, Hamp et al. (2025) used LPA with 398 U.S. families with preschool-aged children and identified two distinct profiles: “social-emotional drivers” who used media to regulate emotions and buffer social situations, and “intentional media” users who planned device use around functional purposes. Although this study focused on younger children and a different media context, it demonstrates that person-centered approaches can reveal qualitatively different orientations toward media that cut across simple quantity-based distinctions.

Despite this progress, all existing LPA studies share two critical limitations: they are based on single-country samples with limited generalizability, and—with the sole exception of Mayen and Camerini (2025)—none have validated the temporal stability of identified profiles using longitudinal data.

### 1.5 Cross-national variation in parental mediation

Ecological systems theory (Bronfenbrenner, 1979) posits that parenting practices are shaped not only by microsystem factors (parent–child relationship quality, family SES) but also by macrosystem influences including cultural norms, national policies, and digital infrastructure. European countries vary substantially in cultural orientations toward child autonomy and parental oversight. Kalmus et al. (2022), analyzing EU Kids Online data from 12 European countries across two time points (2010 and 2018), documented a general trend toward more active mediation and less restrictive mediation over the 8-year period. However, they also found persistent cross-national disparities: Nordic countries consistently favored enabling approaches and post-communist countries maintained higher levels of restriction, reflecting divergent cultural philosophies about children's digital autonomy.

Helsper et al. (2024), analyzing data from eight European countries, found that parental risk perception, digital skills, and prior experience with online risks jointly predicted mediation engagement. They argued that country-level differences in digital infrastructure and parental digital literacy may partly explain why some countries show higher overall mediation intensity. This suggests that the cultural gradient in mediation is not simply about values—it also reflects structural differences in digital environments and parental capabilities.

Liu et al. (2024), in a systematic review of 32 studies from 14 countries, highlighted that family socioeconomic status moderated the link between screen media use and mental health outcomes, with adolescents from lower-SES families facing elevated risks. This finding suggests that cross-national variation in mediation profiles may reflect not only cultural differences but also structural inequalities in access to digital literacy resources.

Despite this evidence of cross-national variation in aggregate mediation practices, no study has examined whether qualitatively distinct mediation profiles emerge consistently across multiple nation-states, or whether profile prevalence varies systematically with cultural context. All existing LPA studies are limited to single-country samples.

### 1.6 The present study and hypotheses

This study addresses three gaps in the literature. First, it applies LPA to data from six European countries simultaneously, testing whether mediation profiles are cross-nationally robust. Second, it uses three-wave longitudinal data to assess the temporal stability of profiles. Third, it links profiles to adolescent wellbeing outcomes. Synthesizing the theoretical and empirical evidence reviewed above, we propose four hypotheses:

**H1 (Profile structure):** based on converging evidence from single-country LPA studies that consistently identify a low–medium–high gradient of mediation intensity (Li et al., 2024; Mayen and Camerini, 2025; Navarro and Jensen, 2026; Sevilla-Fernández et al., 2026), and based on authoritative parenting theory's prediction that warmth and structure co-occur (Baumrind, 1991), we hypothesize that LPA will reveal three to five distinct profiles reflecting a gradient of overall mediation intensity, with restrictive and enabling mediation co-occurring rather than opposing each other.

**H2 (Cross-national variation):** based on ecological systems theory (Bronfenbrenner, 1979) and documented cross-national differences in European parenting practices (Kalmus et al., 2022; Helsper et al., 2024), we hypothesize that profile prevalence will vary significantly across the six countries, reflecting macro-systemic differences in welfare-state regimes, digital infrastructure, and parental digital-literacy resources. Directionally, we expect post-communist Baltic contexts characterized by rapid digital adoption (e.g., Estonia) to show higher Disengaged prevalence, and Mediterranean contexts with stronger familism traditions (e.g., Portugal) to show higher Active All-Round prevalence. Two macro-systemic mechanisms underlie these expectations. First, welfare-state regimes that delegate digital socialization to schools and public infrastructure (e.g., the Nordic-Baltic social-democratic model; Esping-Andersen, 1990) reduce the salience of explicit parental rule-setting. Second, cross-country differences in family digital-literacy infrastructure (Helsper et al., 2024) shift the relative weight of active vs. restrictive strategies. Finland, Germany, Italy, and Poland are expected to occupy intermediate positions consistent with their mixed cultural and welfare-state characteristics.

**H3 (Outcome associations):** based on authoritative parenting theory (Baumrind, 1991), SDT's emphasis on autonomy-supportive structure (Ryan and Deci, 2000), and the meta-analytic finding that positive mediation most strongly predicts positive digital wellbeing (Tan et al., 2025), we hypothesize that the profile combining high restrictive and high enabling mediation will be associated with the most favorable adolescent outcomes (highest life satisfaction, strongest family support, lowest negative affect).

**H4 (Longitudinal stability):** based on developmental theories of adolescent autonomy-seeking (Steinberg, 2001) and Mayen and Camerini's (2025) finding of profile fluctuation over time in a Swiss sample, we hypothesize that profiles will show moderate stability across waves, with a directional trend toward less mediation (disengagement) as adolescents age. The expectation of a directional drift (rather than mere fluctuation) follows from Steinberg's (2001) thesis that adolescent autonomy-seeking systematically reorganizes the parent–child relationship—parents withdraw direct control as children mature, and adolescents progressively reframe parental engagement as intrusive. We refine H4 to specify that this drift may be conditional on macrosystem context: in cultures where parental mediation is normatively framed as developmentally appropriate, the drift may be attenuated or reversed. Country-stratified longitudinal analyses (§3.7) test this refinement.

## 2 Materials and methods

### 2.1 Participants and procedure

Data came from the ySKILLS (Youth Skills) longitudinal survey, a European Union Horizon 2020–funded project (Grant Agreement No. 870612) designed to examine the role of digital skills in youth development (Machackova et al., 2024). The survey was administered in schools across six countries: Estonia (*n* = 1,250), Finland (*n* = 781), Germany (*n* = 1,076), Italy (*n* = 965), Poland (*n* = 1,155), and Portugal (*n* = 1,048). Three waves of data were collected: Wave 1 (Spring 2021), Wave 2 (Spring 2022), and Wave 3 (Spring 2023). Participants were in Grades 6–10 at Wave 1 (age range = 11–20 years, *M* = 14.38, SD = 1.29; 48.1% female; total *N* = 6,275) at Wave 1 (defined as cases with valid W1 age data; analytic subsamples for LPA, EFA, and LTA are reported separately in §2.3). Schools were selected for socioeconomic diversity. Data collection used computer-assisted online questionnaires administered during school hours in 45-min sessions. Informed consent was obtained from children and legal guardians in all countries. The study received ethical approval from institutional review boards in each participating country.

Across the three waves of the ySKILLS panel (*N* = 10,821 unique participants in the full longitudinal dataset), 9,881 participants provided valid responses on at least one parental mediation indicator at one or more waves. All analyses use Mplus 8.3 with FIML for missing data (Enders, 2010), avoiding the selection bias that listwise deletion would introduce given that participants more likely to attrit by Wave 3 differ systematically from those retained (see §4.7 Limitations for the attrition analysis). Analytic sample sizes by analysis: W1 cross-sectional LPA, *N* = 5,675; W1 EFA on the eight mediation items, *N* = 5,833; W2 LPA, *N* = 6,238; W3 LPA, *N* = 5,472; three-wave LTA and multi-group LTA, *N* = 9,881. Among W1 participants with complete gender data, three-wave retention showed a small but statistically detectable gender pattern (χ^2^(3) = 16.70, *p* < 0.001, Cramer's *V* = 0.054): female participants were slightly more likely to complete all three waves (52.4%) than male participants (47.6%). The effect size is practically negligible (*V* < 0.10), consistent with the MAR assumption underlying FIML estimation. Country representation was preserved: Estonia (W1 *N* = 1,016), Finland (533), Germany (951), Italy (850), Poland (823), Portugal (944).

### 2.2 Measures

***Restrictive mediation. Three*** items assessed parents' rule-setting behaviors: “Sets rules about when you can use the internet,” “Forbids you from doing certain things online (e.g., play certain online games, use some websites),” and “Sets rules about the total time you can spend online in a day.” All items were rated on a five-point scale (1 = *not at all true* to 5 = *very true*). Composite scores were computed as the mean of all three items. Internal consistency was good: Cronbach's α = 0.84 at Wave 1 (range across countries: 0.81–0.87), 0.85 at Wave 2, and 0.86 at Wave 3.

***Enabling mediation*. ***Four* items measured communicative engagement: “Suggests ways to use the internet safely,” “Talks to you about what you do on the internet,” “Encourages you to explore and learn things on the internet,” and “Helps you when something bothers you on the internet.” Items used the same five-point scale. Composite scores were computed as the mean of at least three valid items. Internal consistency was good: α = 0.81 at Wave 1 (range: 0.74–0.83), 0.81 at Wave 2, and 0.82 at Wave 3.

***Monitoring (external validation variable)***. A single item assessed monitoring: “Checks/controls what you do on the internet” (same five-point scale). This variable was reserved as an external validation measure and was *not* included as an indicator in the LPA, following the strategy of validating profiles against theoretically related but methodologically independent measures.

***Co-use (not assessed)*. **Although co-use is one of the four parental mediation practices outlined in Section 1.1, the ySKILLS three-wave battery did not include co-use items. The present analysis is therefore restricted to restrictive and enabling (active) mediation as perceived by the child, with the single monitoring item reserved for external validation. This instrumentation constraint is acknowledged in Section 4.7.

***Adolescent outcomes. Six*** outcome variables were assessed at Wave 1: (1) positive life satisfaction, a multi-item composite (four-point scale; *M* = 3.37, SD = 0.69); (2) negative affect (four-point scale; *M* = 2.40, SD = 0.89); (3) family support, measuring perceived family relationship quality (four-point scale; *M* = 3.50, SD = 0.60); (4) friend support (four-point scale; *M* = 3.25, SD = 0.73); (5) self-efficacy (four-point scale; *M* = 2.95, SD = 0.65); and (6) sensation seeking (five-point scale; *M* = 3.18, SD = 0.93). All measures were administered at all three waves.

### 2.3 Analytic strategy

Following best-practice recommendations for LPA (Nylund-Gibson and Choi, 2018; Spurk et al., 2020), two standardized composite scores—restrictive and enabling mediation—served as continuous indicator variables. Gaussian mixture models with full covariance matrices were estimated for one through seven profiles, each with 200 random starting values and 1,000 maximum iterations to avoid local maxima.

Prior to LPA, we examined the convergent and discriminant validity of the eight mediation items (three restrictive, four enabling, one monitoring) using exploratory factor analysis in Mplus 8.3 (2012-2018 Muthen & Muthen) with robust maximum likelihood (MLR) estimation and GEOMIN oblique rotation (the Mplus default for oblique factor analysis); *N* = 5,833 with FIML across cases having any valid W1 mediation indicator. Sampling adequacy and sphericity were evaluated on the pairwise correlation matrix using the Kaiser–Meyer–Olkin (KMO) statistic and Bartlett's test of sphericity (computed via R psych on the listwise-complete subsample, *N* = 4,462). The KMO statistic was 0.846 (overall; per-item MSAs ranged 0.777–0.895, all above the 0.70 threshold), and Bartlett's test was highly significant, χ^2^(28) = 14,651.70, *p* < 0.001, indicating that the correlation matrix was suitable for factor analysis. The number of factors to retain was determined by comparison of one- through four-factor solutions against Hu and Bentler (1999) fit-index cutoffs and confirmed via Horn's parallel analysis (R psych::fa.parallel, 1,000 random datasets, 95th-percentile criterion). This analysis was conducted in response to concerns about the empirical distinctiveness of restrictive and enabling mediation given their bivariate correlation of *r* = 0.40.

Model selection was guided by multiple criteria: the Bayesian Information Criterion (BIC; lower values preferred), entropy (values above 0.80 indicating good classification quality), the minimum class proportion (above 5% for interpretability), and substantive interpretability (Nylund-Gibson and Choi, 2018). As Spurk et al. (2020) emphasized, statistical fit indices should be weighed against theoretical considerations, and entropy should not serve as a sole selection criterion.

The selected profiles were externally validated against the held-out monitoring variable. Cross-national distributions were tested using chi-square tests with adjusted standardized residuals (ASR; |*z*| > 1.96 = statistically significant) and Cramér's *V* as the effect-size metric. Profile differences on the six distal wellbeing outcomes were assessed via the Bolck–Croon–Hagenaars (BCH) auxiliary-variable method (Bolck et al., 2004), which corrects for classification uncertainty when comparing outcome means across latent classes. Longitudinal stability was evaluated via model-estimated transition probability matrices and Cohen's κ for class-membership agreement across waves. Primary latent-variable analyses (EFA, LPA, three-wave LTA, multi-group LTA with country as a known-class variable, BCH distal-outcome estimation, and age-moderated LTA) were conducted in Mplus 8.3 (Muthén and Muthén, 1998–2017) with robust maximum likelihood (MLR) estimation and full-information maximum likelihood (FIML) for missing data (Enders, 2010); 500 random starts were used for all mixture models to guard against local maxima. Auxiliary analyses—descriptive statistics, χ^2^ and Cramér's *V* tests, attrition diagnostics, parallel analysis (R psych::fa.parallel; Revelle, 2026), and the supplementary *k*-means comparison—were conducted in R 4.5.2 (R Core Team, 2025). We acknowledge upfront that formal tests of measurement invariance (configural, metric, scalar) of the mediation items across countries were not feasible with two-indicator latent factors and are not provided by ySKILLS documentation. Cross-national comparisons are therefore interpreted under the assumption — rather than the demonstration—of measurement invariance, an explicit constraint that we revisit in the Limitations.

## 3 Results

### 3.1 Descriptive statistics

Table 1 presents descriptive statistics and bivariate correlations. Restrictive and enabling mediation were moderately correlated (*r* = 0.40), and both correlated substantially with monitoring (*r* = 0.52 and 0.51), supporting the theoretical expectation that mediation strategies co-occur.

**Table 1:** Descriptive statistics and bivariate correlations.

Variable	*N*	*M*	SD	1	2	3	4	5	6
1. Restrictive	5,311	2.59	1.16	—					
2. Enabling	5,481	2.75	1.02	0.40	—				
3. Monitoring^†^	5,486	2.13	1.20	0.52	0.51	—			
4. Satisf. (+)	5,265	3.37	0.69	0.09	0.19	0.08	—		
5. Neg. affect	5,376	2.40	0.89	−0.08	−0.09	−0.06	−0.54	—	
6. Family supp.	5,652	3.50	0.60	0.05	0.24	0.06	0.45	−0.38	—

All |*r*| ≥ 0.05 significant at *p* < 0.01. Ms and SDs computed on FIML per-variable samples (*N*s shown in the *N* column). Correlations computed on pairwise complete observations; off-diagonal pairwise *N*s range from 4,745 to 5,295. The LPA analytic sample is *N* = 5,675 (Mplus FIML on the two LPA indicators: restrictive and enabling).

^†^Validation variable, not included in LPA.

Convergent/discriminant validity. EFA conducted in Mplus 8.3 (MLR estimator, GEOMIN oblique rotation, *N* = 5,833 with FIML; Wave 1 mediation items) confirmed two factors. The one-factor solution fit poorly (RMSEA = 0.198, CFI = 0.655, χ^2^(20) = 4,598.22, *p* < 0.001); the two-factor solution fit excellently (RMSEA = 0.058, CFI = 0.981, TLI = 0.959, SRMR = 0.019, χ^2^(13) = 267.62, *p* < 0.001); the three-factor solution failed to converge in Mplus, and the four-factor solution was overfit (df = 2). Horn's parallel analysis (1,000 random datasets) indicated three factors under the strict eigenvalue criterion, but the empirical third factor reflected only the single monitoring item; combined with the three-factor non-convergence in Mplus, the two-factor structure was retained (Fabrigar et al., 1999; Hayton et al., 2004). The two-factor GEOMIN oblique pattern matrix showed clean simple structure: restrictive items loaded 0.619–0.874 on F1 with negligible cross-loadings (0.005–0.154); enabling items loaded 0.653–0.783 on F2 with negligible cross-loadings (0.009–0.119); the single monitoring item loaded moderately on both F1 (0.305) and F2 (0.443)—consistent with monitoring as a hybrid indicator drawing on both control-oriented and engagement-oriented parenting. Factor correlation was Φ = 0.493, indicating substantial but non-redundant overlap. Restrictive and enabling mediation are therefore empirically distinct dimensions, not indicators of a single latent variable.

### 3.2 Model selection

**Figure 1:**
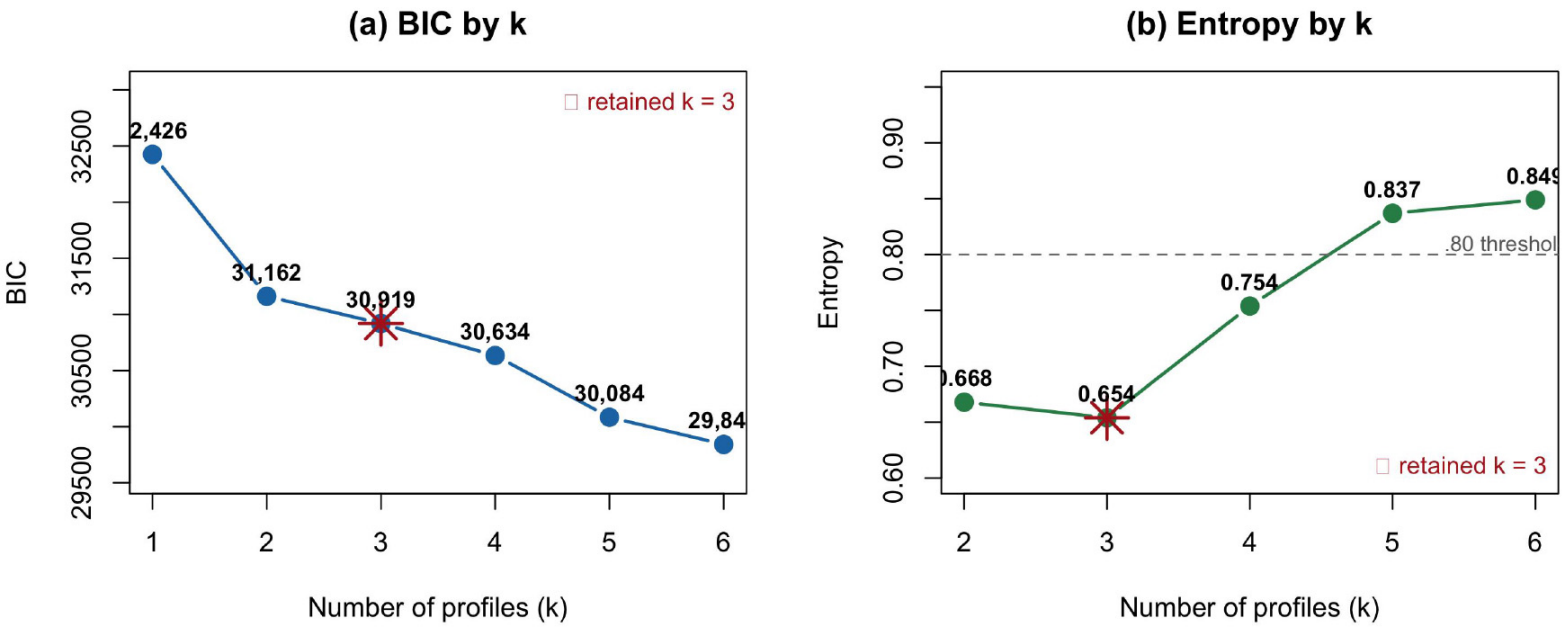
Model selection: BIC **(a)** and entropy **(b)** for 1–6 profiles.

Table 2 presents Mplus 8.3 FIML fit indices for *k* = 1 through *k* = 6 profile solutions. BIC decreased monotonically from *k* = 1 (32,426) through *k* = 6 (29,842), with no clear elbow. While *k* = 4 (BIC = 30,634, entropy = 0.754, minimum class = 13.6%) and higher-k solutions had progressively lower BIC values, several considerations led us to retain *k* = 3 as the substantively preferred and most stable model: (a) convergence stability—all 100 final-stage random starts replicated the best log-likelihood for *k* = 2 and *k* = 3, whereas only 24 of 100 reached the global maximum at *k* = 4, 11 of 100 at *k* = 5, and 1 of 100 at *k* = 6, indicating that solutions beyond *k* = 3 are increasingly ill-conditioned and unstable; (b) substantive interpretability—the *k* = 4 fourth class did not produce the configural profile (e.g., authoritarian: high-restriction/low-enabling) that would constitute the substantive test of greater complexity; instead it split the high-mediation end of the same intensity gradient into two adjacent sub-clusters; (c) mechanical BIC reduction on continuous indicators—as Bauer (2007) noted, BIC reductions on continuous LPA indicators can arise from finer-grained slicing of an underlying gradient rather than from discovery of new latent subgroups, consistent with our observation that *k* = 5 and *k* = 6 continue to lower BIC while producing classes as small as 6.7%; and (d) class-size adequacy—the *k* = 3 solution retains a minimum class proportion of 24.4%, well above conventional 5% thresholds (Nylund-Gibson and Choi, 2018), while preserving strong external validation against the held-out monitoring variable (reported below). Its entropy (0.654) fell below the 0.80 threshold; however, Spurk et al. (2020) cautioned that entropy reflects classification precision rather than profile validity, and Nylund-Gibson and Choi (2018) advised against using entropy as a sole criterion. See Figure 1 for the BIC and entropy curves across all *k* = 1–6 solutions.

**Table 2:** Mplus 8.3 FIML model fit indices for 1–6 profile solutions.

K	LL	BIC	Entropy	Min. class (%/LL repl.)
1	−16,195.71	32,426	—	100.0%/n/a
2	−15,550.54	31,162	0.668	41.4%/100
3^*^	−15,416.35	30,919	0.654	24.4%/100
4	−15,260.92	30,634	0.754	13.6%/24
5	−14,972.84	30,084	0.837	7.6%/11
6	−14,838.70	29,842	0.849	6.7%/1

*N* = 5,675 (Mplus FIML on W1 LPA indicators: restrictive and enabling; STARTS = 500 100).

^*^ Selected model. LL, log-likelihood; Entropy reflects classification precision; Min. class, smallest estimated class proportion from the model-implied posterior; LL repl., number of final-stage random starts (out of 100) that converged to the best log-likelihood (n/a for *k* = 1, which is a single-class baseline). The monotonic BIC decrease combined with precipitously declining LL replication (100 → 100 → 24 → 11 → 1) supports retaining *k* = 3 as the largest stable solution.

### 3.3 Profile characterization and external validation

**Figure 2:**
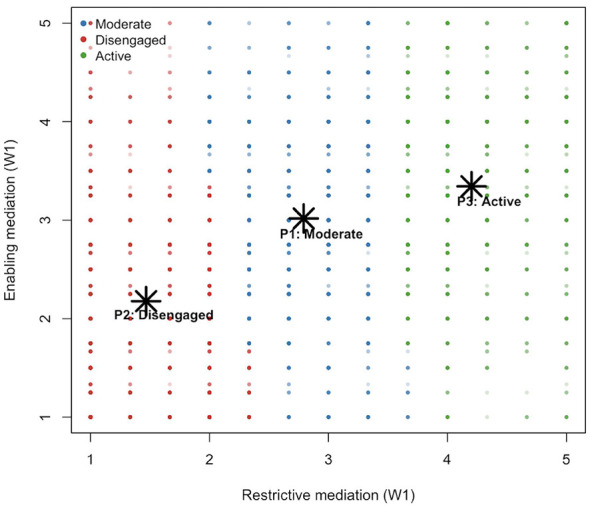
Scatter plot of parental mediation profiles. Stars indicate centroids.

The three profiles formed a clear gradient of mediation intensity (Figure 2; Table 3), supporting H1. Profile 1 (Disengaged; 40.2% of W1 sample) showed the lowest restrictive mediation (*M* = 1.50, SD = 0.53) and enabling mediation (*M* = 2.23, SD = 0.91). Profile 2 (Moderate Balanced; 35.4%) showed intermediate values (restrict *M* = 2.77, SD = 0.53; enable *M* = 2.93, SD = 0.91). Profile 3 (Active All-Round; 24.4%) showed the highest values (restrict *M* = 4.13, SD = 0.53; enable *M* = 3.33, SD = 0.91). Mplus 8.3 LPA used the class-invariant variance parameterization (variances constrained equal across classes; restrict variance = 0.282, enable variance = 0.834); convergence was stable with 500 random starts all replicating the best loglikelihood.

**Table 3:** Profile characterization.

Profile	n (%)	Restrict. M(SD)	Enabling M(SD)	Monitor. M(SD)^†^	Avg. PP
P1: disengaged	2,283 (40.2%)	1.50 (0.53)	2.23 (0.91)	1.50 (0.82)	0.865
P2: moderate	2,008 (35.4%)	2.77 (0.53)	2.93 (0.91)	2.24 (1.10)	0.766
P3: active	1,383 (24.4%)	4.13 (0.53)	3.33 (0.91)	3.04 (1.26)	0.866

^†^Not included in LPA. Avg. PP, average posterior probability.

External validation was strong. Profiles differed significantly on monitoring: *F*(2, 5,410) = 910.80, *p* < 0.001, η^2^ = 0.252. Monitoring increased monotonically across profiles (Disengaged: *M* = 1.50, SD = 0.82; Moderate: *M* = 2.24, SD = 1.10; Active: *M* = 3.04, SD = 1.26). Pairwise Cohen's d values for monitoring ranged from 0.69 (Moderate vs. Active) to 1.54 (Disengaged vs. Active), spanning medium-to-large to very-large effects, confirming that profiles captured a meaningful latent dimension along the intensity gradient.

To probe the robustness of this gradient structure against modeling choices, we conducted a complementary cluster analysis using *k*-means (Hartigan–Wong algorithm; 20 random starts) on the same standardized indicators (*N* = 5,117 complete cases on restrictive and enabling mediation). Overall classification agreement between the *k*-means solution and the Mplus LPA modal-class assignment was 63.8%. Profile-specific row agreement was 98.8% for Active All-Round, 68.1% for Disengaged, but only 34.8% for Moderate Balanced; *k*-means reassigned the remaining LPA-Moderate cases roughly equally to Disengaged (22.1%) and Active (43.1%). The extreme profiles are thus robust to method choice, whereas the Moderate Balanced profile is method-dependent. This pattern is consistent with the general psychometric observation that, when indicators are substantially correlated, the middle of an intensity gradient is the region where person-oriented methods exhibit the greatest classification uncertainty (Bauer, 2007).

### 3.4 Cross-national profile distribution

**Figure 3:**
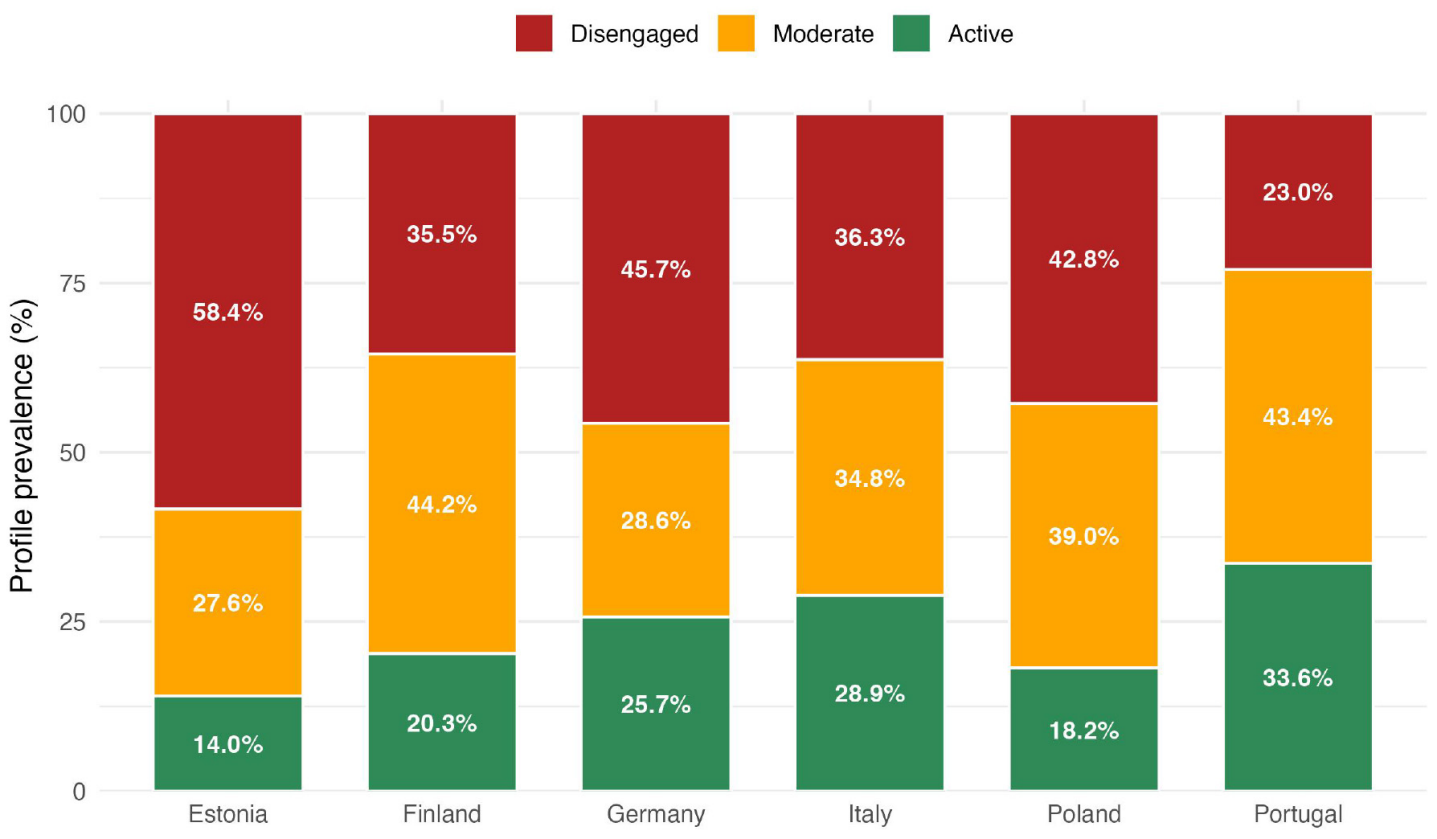
Profile distribution by country (%). Darker shading = higher prevalence.

H2 was supported (see Figure 3): profile prevalence varied significantly across the six countries, χ^2^(10) = 365.63, *p* < 0.001, Cramér's *V* = 0.179, indicating a small-to-medium effect (Cohen, 1988). Adjusted standardized residuals (Agresti, 2018) identified Estonia as having the highest Disengaged proportion (58.4%; ASR = +13.41) and the lowest Active All-Round proportion (14.0%; ASR = −8.48); Portugal showed the opposite pattern (Disengaged 23.0%, ASR = −12.95; Active All-Round 33.6%, ASR = +8.49). This cross-national gradient aligns with the macro-systemic framework outlined in §1.5: country-level differences in cultural norms, welfare-state regimes (Esping-Andersen, 1990), and digital infrastructure (Helsper et al., 2024) appear to jointly shape the prevalence of disengagement and the cultural acceptability of intensive parental involvement. Estonia, a post-communist Baltic state characterized by rapid digital adoption, showed the highest Disengaged prevalence; Portugal, where Mediterranean familism traditions persist alongside relatively lower aggregate digital literacy resources, showed the highest Active All-Round prevalence. Finland, Germany, Italy, and Poland occupied intermediate positions consistent with their mixed cultural and welfare-state characteristics. This empirical configuration is consistent with Kalmus et al.'s (2022) finding that European mediation patterns reflect persistent contextual variation rather than convergence.

### 3.5 Profile differences on adolescent outcomes

H3 was supported overall, though pairwise contrasts revealed a threshold rather than gradient pattern. Using the Bolck–Croon–Hagenaars (BCH) auxiliary-variable method (Asparouhov and Muthén, 2014), we estimated class-specific outcome means weighted by posterior class-membership probability and tested equality across classes via Wald χ^2^. Overall tests were significant for all six adolescent outcomes (Table 4; all overall χ^2^(2) ≥ 8.29, *p* < 0.05). Active All-Round profile members reported the highest positive life satisfaction (*M* = 3.47, SE = 0.02), the strongest family support (*M* = 3.57, SE = 0.02), and the lowest negative affect (*M* = 2.27, SE = 0.03). Sensation seeking showed a reverse gradient: Disengaged profile members scored highest (*M* = 3.32, SE = 0.02). Importantly, however, pairwise BCH contrasts revealed that the Disengaged–vs–Active contrast was significant on every outcome (all *p* < 0.05), whereas Moderate–vs–Active contrasts were non-significant for positive life satisfaction (*p* = 0.229), family support (*p* = 0.113), friend support (*p* = 0.279), and sensation seeking (*p* = 0.159). This threshold pattern—substantial differences between Disengaged and the two higher intensity profiles, but smaller differences between Moderate and Active—suggests that the wellbeing benefit accrues primarily from moving out of disengagement rather than from intensifying engagement above moderate levels, complicating a simple “more mediation is better” interpretation (see §4.3).

**Table 4:** Profile differences on six adolescent outcomes (Mplus BCH method).

Outcome	P1 M(SE)	P2 M(SE)	P3 M(SE)	*χ^2^*(2)	Pattern
Satisf. (+)	3.26 (0.02)	3.43 (0.02)	3.47 (0.02)	60.83^***^	*D*<*M* = *A*
Neg. affect	2.48 (0.02)	2.41 (0.03)	2.27 (0.03)	32.04^***^	*D* = *M* > *A*
Family supp.	3.45 (0.02)	3.53 (0.02)	3.57 (0.02)	28.20^***^	*D*<*M* = *A*
Friend supp.	3.22 (0.02)	3.26 (0.02)	3.31 (0.03)	8.29^*^	*D*<*A* only
Self-efficacy	2.94 (0.02)	2.89 (0.02)	3.04 (0.02)	24.15^***^	*D* = *M*<*A*
Sens. seeking	3.32 (0.02)	3.12 (0.03)	3.06 (0.03)	54.68^***^	*D* > *M* = *A*

Means and standard errors from the Bolck–Croon–Hagenaars (BCH) auxiliary-variable method (Asparouhov and Muthén, 2014) with *N* = 5,675 (W1 LPA FIML base). χ^2^(2) is the overall BCH Wald equality-of-means test across the three latent classes. The “Pattern” column summarizes significant pairwise BCH contrasts: “ < ”, “>”, and “=” connect classes whose pairwise Wald χ^2^(1) test was significant (*p* < 0.05) or non-significant, respectively. P1 = Disengaged, P2 = Moderate Balanced, P3 = Active All-Round. ^*^p < 0.05; ^***^p < 0.001.

### 3.6 Longitudinal stability

**Figure 4:**
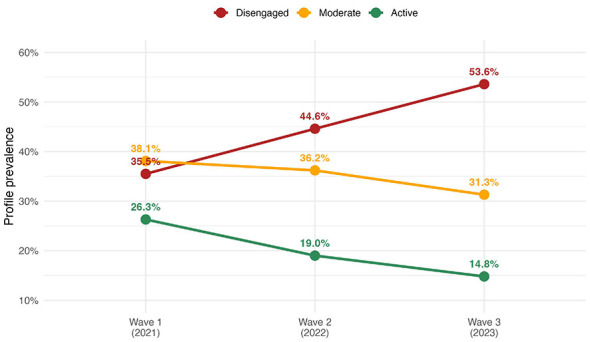
Profile prevalence across three waves.

H4 was supported (see Figure 4). The three-class LTA estimated in Mplus 8.3 with measurement invariance constraints across waves (class means anchored: Disengaged restrict *M* = 1.37, enable *M* = 2.12; Moderate Balanced 2.62, 2.84; Active All-Round 4.06, 3.29) yielded smooth, stable parameter estimates (500 random starts all replicated the best loglikelihood; LL = −61,962 [multi-group model], BIC = 124,485). The model-implied Disengaged prevalence rose from 35.5% (W1) to 44.6% (W2) to 53.6% (W3); Moderate Balanced declined modestly from 38.1% to 36.2% to 31.3%; Active All-Round declined from 26.3% to 19.0% to 14.8%. Estimates of the W1 distribution differ slightly from the cross-sectional LPA above because the LTA uses full-information maximum likelihood (FIML) across all three waves (*N* = 9,881), incorporating participants who provided data at any of W1, W2, or W3.

Transition matrices (Table 5) revealed Disengaged as a highly absorbing state (W1 → W2 stability = 92.8%; W2 → W3 = 93.3%), Moderate with intermediate stability (66.8%; 63.2%), and Active All-Round as least stable (58.7%; 58.4%). Wave-to-wave Cohen's κ coefficients were moderate-to-substantial (κ = 0.599 for W1 → W2; 0.607 for W2 → W3); the 2-year W1 → W3 κ was lower (0.364) reflecting cumulative reassignment across both transitions (Landis and Koch, 1977). Downward transitions (toward lower mediation intensity) exceeded upward transitions by approximately 4:1, supporting the directional drift toward Disengagement hypothesized in H4. The near-identity of the W1 → W2 and W2 → W3 matrices indicated stationary transition dynamics.

**Table 5:** Mplus 3-wave LTA transition probabilities (W1 → W2 and W2 → W3) with measurement invariance across waves.

W1→W2 transitions (*κ* = 0.599)			
	W2 P1	W2 P2	W2 P3
W1 P1: Disengaged	92.8%	4.9%	2.3%
W1 P2: moderate	25.9%	66.8%	7.3%
W1 P3: active	7.0%	34.3%	58.7%
**W2→W3 transitions (κ = 0.607)**			
	W3 P1	W3 P2	W3 P3
**W2 P1: Disengaged**	93.3%	4.5%	2.1%
**W2 P2: moderate**	29.0%	63.2%	7.8%
**W2 P3: active**	7.7%	33.9%	58.4%

*N* = 9,881 (Mplus FIML 3-wave LTA, 07_lta_3wave_invariance.out). Bold diagonal = stability (probability of remaining in the same profile across the wave). Cohen's κ =.599 for W1 → W2; 0.607 for W2 → W3; 0.364 for cumulative W1 → W3, indicating moderate-to-substantial classification agreement across adjacent waves and cumulative reassignment across the two-year window. Country-specific transition matrices are provided in Table 6.

Age-moderation analysis. To test whether the observed drift reflects developmental aging (Steinberg, 2001) rather than period effects or measurement artifacts, we re-estimated the LTA with W1 age (mean-centered at 14.42 years) as a covariate of W1 class membership and of W1 → W2 and W2 → W3 transition probabilities. Reference class was Disengaged. Age strongly predicted W1 baseline class: each additional year was associated with 0.529-fold odds of being in Active All-Round vs. Disengaged (*p* < 0.001) and 0.666-fold odds of being in Moderate Balanced vs. Disengaged (*p* < 0.001) — i.e., older adolescents at baseline were markedly more likely to be classified as Disengaged. Controlling for prior-wave class, age also significantly predicted transitions away from Active All-Round: each additional year was associated with 0.773-fold odds of remaining in or transitioning to Active vs. Disengaged at W2 (*p* < 0.001) and 0.820-fold odds at W3 (*p* = 0.021). Transitions involving Moderate Balanced were not significantly moderated by age beyond what prior class explained (both *ps* > 0.25). The pattern indicates that age primarily shapes baseline class membership, while additional age-related drift is concentrated at the high-mediation end of the gradient: as adolescents mature, parents of those previously engaged at the Active All-Round level are particularly likely to disengage.

### 3.7 Country-stratified longitudinal trajectories

Reviewer feedback prompted us to disaggregate transition probabilities by country via a formal multi-group Latent Transition Analysis in Mplus 8.3, with country as a known class variable (KNOWNCLASS) allowing W1 profile distribution and W1 → W2 and W2 → W3 transition probabilities to vary by country, with measurement invariance constraints on profile centers. Convergence was stable (500 random starts; 61 free parameters; AIC = 124,046; BIC = 124,485). Contrary to preliminary listwise-based analyses, the multi-group FIML LTA revealed UNIFORM drift toward Disengaged across all six countries from W1 to W3: net 2-year increases in Disengaged prevalence ranged from +12.1 pp in Portugal to +25.2 pp in Italy (Estonia + 21.6; Finland + 18.6; Poland + 13.5; Germany + 12.8), with corresponding declines in Active All-Round prevalence in every country. Country-specific transition matrices confirmed Disengaged as a highly absorbing state in every context (one-wave stability range: 0.830 in Portugal to 0.965 in Estonia). The discrepancy with our earlier listwise-based analyses (preliminary Python analyses, and comparable patterns in Mayen and Camerini, 2025) reflects differential attrition: Disengaged participants at W1 were more likely to drop out by W3, biasing listwise transition analyses toward apparent stability or counter-drift in countries with high attrition. The FIML LTA incorporates all participants under the MAR assumption and recovers the underlying universal trajectory toward Disengagement during early-to-middle adolescence.

The multi-group LTA with country as a known class variable allowed direct estimation of country-specific W1 distributions, W1 → W2 transitions, and W2 → W3 transitions. Cross-country variation in transition probabilities was substantial (e.g., one-wave Moderate → Disengaged transition probability ranged from 0.143 in Portugal to 0.441 in Estonia), but the directional pattern was uniform: all six countries showed net W1 → W3 movement toward Disengaged and away from Active All-Round. Country differences therefore reflect speed and depth of drift rather than direction. Full country-specific stability and Disengaged-shift metrics are provided in Table 6.

**Table 6:** Country-specific transitions: Disengaged absorbing-state stability and net W1 → W3 Disengaged increase.

Country	W1 → W2 D → D	W2 → W3 D → D	W1 → W3 Δ Dis. (pp)
Estonia	96.5%	95.6%	+21.6
Finland	92.4%	89.6%	+18.6
Germany	92.8%	91.0%	+12.8
Italy	94.0%	95.0%	+25.2
Poland	89.5%	90.2%	+13.5
Portugal	83.0%	83.0%	+12.1

*N* = 9,881 (Mplus multi-group FIML LTA, 08_multigroup_lta.out, KNOWNCLASS = country). D → D = probability of remaining Disengaged across the wave transition. Net Δ Disengaged = country's W1 Disengaged percentage subtracted from its W3 Disengaged percentage. All six countries show monotonic increase in Disengaged prevalence; Disengaged is a highly absorbing state in every country (≥ 83% one-wave stability).

## 4 Discussion

This study was among the first to provide cross-national, longitudinal evidence for a typology of parental digital mediation. Three profiles—Disengaged, Moderate Balanced, and Active All-Round—emerged from data spanning six European countries and three annual waves, with all four hypotheses largely supported. Below, we discuss these findings in dialogue with existing research and theory.

### 4.1 Intensity vs. configuration: a conceptual reflection

The three-profile gradient (H1 supported) converges strikingly with single-country findings across diverse cultural contexts. Mayen and Camerini (2025) identified three classes in Switzerland (Hands-off, Moderate, Enforcing and Engaged); Navarro and Jensen (2026) found a four-profile gradient in the United States (Uninvolved through High Mediators); Li et al. (2024) observed coordinated low-level and high-level mediation in China; and Sevilla-Fernández et al. (2026) identified four profiles in Spain ranging from minimal to integral mediation. The convergence of a low–medium–high involvement gradient across such diverse cultural, linguistic, and policy contexts suggests that this structure is a cross-culturally robust feature of digital parenting—not an artifact of any particular sample or measurement approach.

A theoretically important finding is the absence of a “restrictive-only” profile—parents who impose strict rules without communicative support. In our data, restrictive and enabling mediation were positively coupled (*r* = 0.40), and no profile showed high restriction paired with low enabling. This diverges from Sevilla-Fernández et al. (2026), who identified a “technological mediation” profile in Spain characterized by higher technical controls relative to communicative strategies. The discrepancy likely reflects measurement differences: our two-dimensional measure could not distinguish technology-focused from other restrictive strategies, whereas their six-dimensional instrument could. Nevertheless, the co-occurrence of restriction and enabling in our data is consistent with authoritative parenting theory (Baumrind, 1991): in this sample, rule-setting was embedded within a communicative relational context rather than operating as isolated behavioral control. Navarro and Jensen (2026) observed a similar pattern, noting that their profiles.

“*mirrored general parenting styles to a limited degree*” (p. 481)

supporting authoritative and uninvolved patterns but not authoritarian (high restriction, low enabling) ones. Two theoretically distinct conceptualizations of mediation could in principle be captured: (a) perceived intensity of parental engagement as an aggregate frequency measure across strategies, and (b) situation-specific variation in parental behavior and the breadth of strategies actually deployed. The ySKILLS items measure perceived frequency and so target the first conceptualization. Whether qualitatively distinct configurations (e.g., high-restriction/low-enabling vs. low-restriction/high-enabling) would emerge under a broader, situation-specific instrument such as Sevilla-Fernández et al.'s (2026) six-dimensional measure remains an open empirical question. Our intensity-based gradient should therefore be read as the result that this measurement model can produce, not as a refutation of more configural typologies of mediation. We qualify this consistency, however: Baumrind's framework also predicts authoritarian (high-restrict/low-enable) and indulgent (low-restrict/high-enable) configurations, which our solution did not recover. The present data therefore corroborate the high–high (authoritative) and low–low (uninvolved) ends of the Baumrind typology but do not provide configural evidence for the full four-fold typology. Whether such configural profiles would emerge under richer instrumentation—for example, a six-dimensional mediation measure (Sevilla-Fernández et al., 2026) explicitly designed to disambiguate restriction from enablement—remains a question for future work.

The finding that mediation intensity—rather than mediation configuration—primarily distinguished the profiles has implications for how we conceptualize digital parenting. Sevilla-Fernández et al. (2026) reached a similar conclusion:

“*the amount of mediation is more relevant than the specific strategy*” (p. 491).

If confirmed in future studies with broader measurement, this would suggest that the field's traditional emphasis on identifying the “right” strategy may be secondary to promoting overall parental engagement in children's digital lives.

### 4.2 Cross-national patterning: welfare-state regimes and digital infrastructure

The cross-national distribution (H2 supported) followed a clear macro-systemic gradient. Estonia, a digitally advanced post-communist Baltic state, had the highest Disengaged proportion (58.4%; ASR = +13.41) and the lowest Active All-Round proportion (14.0%; ASR = −8.48). Portugal, where Mediterranean familism traditions persist alongside relatively lower aggregate digital literacy resources (Helsper et al., 2024), had the highest Active All-Round prevalence (33.6%; ASR = +8.49) and one of the lowest Disengaged proportions (23.0%; ASR = −12.95). This pattern extends Kalmus et al.'s (2022) observation that Nordic and post-Soviet countries favor less restrictive parenting while Catholic Southern European countries maintain closer parental oversight, situating the variation within the welfare-state regime and digital-infrastructure differences emphasized in §1.5.

Finland presented an intriguing case. Despite cultural similarities to Estonia in many respects, it showed disengagement (35.5%) close to the cross-national average paired with a high Moderate Balanced share (44.2%). This may reflect Finland's internationally recognized educational system and its structured approach to digital literacy promotion, which encourages parental engagement through institutional support rather than relying solely on individual parenting decisions. In contrast, Estonia's highly digitalized society—where digital services are ubiquitous and children navigate online spaces with relative independence—may normalize lower parental oversight. Helsper et al. (2024) found that parents with stronger digital skills engaged in more mediation; Finland's emphasis on structured digital education may produce parents who are both digitally skilled and institutionally encouraged to mediate, even within a culturally autonomy-supportive framework.

Poland's high Moderate Balanced rate (39.0%) and low Active All-Round rate (18.2%) present another interesting case. As a post-communist country undergoing rapid digitalization, Poland may be transitioning between traditional restrictive norms and newer enabling approaches, resulting in a concentration of moderate, balanced mediation. These nuanced country-level patterns suggest that single cultural-dimension indices capture only part of the picture; national digital policies, educational traditions, and the pace of digital transformation all shape the mediation landscape.

### 4.3 Comprehensive mediation and adolescent wellbeing

H3 was supported: the Active All-Round profile—combining high enabling with high restrictive mediation—was consistently associated with the most favorable outcomes across all domains. This finding converges with multiple lines of evidence. Tan et al.'s (2025) meta-analysis concluded that

“*the association between digital parenting and children's positive digital wellbeing was stronger for positive mediation than negative mediation or co-use*” (p. 1).

Li et al. (2024) found that coordinated high-level mediation predicted the lowest problematic media use. Sevilla-Fernández et al. (2026) found that integral mediation (high across all six dimensions) was associated with the lowest screen time. Together, these converging results from person-centered studies across Western European, Southern European, U.S., and Chinese contexts suggest that comprehensive parental engagement—not any single strategy in isolation—is most beneficial.

From an SDT perspective (Ryan and Deci, 2000), the Active All-Round profile may succeed because it simultaneously satisfies children's needs for structure (through clear rules), competence (through guidance on safe and productive internet use), and relatedness (through open communication about digital experiences). This interpretation aligns with Mayen and Camerini (2025) counterintuitive finding that the “Moderate”—not the “Hands-off”—class reported the lowest perceived autonomy in their Swiss sample. Comprehensive, clearly communicated mediation may paradoxically support greater perceived autonomy than ambiguous, inconsistent mediation, because clear boundaries provide a framework within which adolescents can exercise meaningful choice.

The finding that Disengaged adolescents scored highest on sensation seeking raises an important interpretive question about the direction of influence between adolescent temperament and parental mediation. Rather than reflecting parental neglect, the Disengaged profile may partly capture adolescents who actively resist mediation—a developmentally normative process of autonomy-seeking (Steinberg, 2001). Alternatively, parents of high-sensation-seeking adolescents may reduce mediation efforts after finding them ineffective. Chen and Shi (2019) found that age significantly moderated mediation effects, with strategies becoming less effective as children entered adolescence. This bidirectional dynamic—where both parent and child behaviors shape the mediation environment—cannot be fully disentangled with the present cross-sectional outcome analysis and represents an important direction for future longitudinal mediation modeling.

Effect sizes for outcome comparisons were small to moderate (η^2^ = 0.008–0.023). This is consistent with the broader literature: Milosevic et al. (2022), analyzing EU Kids Online data from 16 countries, found that family environment and SES accounted for 43% of variance in life satisfaction, while enabling mediation accounted for only 1%. Parental mediation is one of many factors—alongside peer relationships, school environment, socioeconomic resources, and individual temperament—that shape adolescent wellbeing. Small effects at the individual level can nevertheless translate into meaningful population-level impacts when aggregated across millions of families.

### 4.4 Developmental trajectories: the drift toward disengagement

The longitudinal analyses (H4 supported) revealed a clear developmental trajectory: Disengaged prevalence grew from 35.5% to 53.6% over 2 years, while Active All-Round declined from 26.3% to 14.8%. This pattern is consistent with normative theories of adolescent development (Steinberg, 2001), which posit that adolescents increasingly seek autonomy from parental oversight as they mature. Mayen and Camerini (2025), the only prior longitudinal LPA study of parental mediation, found similar fluctuation in their Swiss sample, with adolescents moving between Enforcing and Engaged and Moderate classes across three waves.

Our study extends their finding to a larger, cross-national sample and identifies a novel structural feature: the directional asymmetry of transitions. Transitions were predominantly downward—from Active All-Round to Moderate, and from Moderate to Disengaged—with very few upward transitions. Only 2.1–2.3% of Disengaged parents transitioned directly to Active All-Round between consecutive waves (pooled 3-wave LTA: W1 → W2 = 2.3%, W2 → W3 = 2.1%). This identifies the Disengaged profile as an “absorbing state”: once parents adopt a hands-off approach, re-engagement is rare. This asymmetry has not been previously documented in the parental mediation literature and has important implications for intervention design.

Country-specific multi-group LTA (§3.7) confirmed that the directional drift toward Disengagement was uniform across all six countries, though magnitude varied substantially (2-year net Disengaged increases ranged from +12 pp in Portugal to +25 pp in Italy). This pattern strengthens rather than qualifies the developmental account: parents across diverse European contexts progressively withdraw from active digital mediation as adolescents mature, with the speed of withdrawal varying by macrosystem context but the direction invariant. Mediterranean (Italy, Portugal) and Baltic (Estonia) contexts showed the most rapid drift; Central European (Germany, Poland) and Nordic (Finland) contexts showed slower but consistent drift. Likely contributors include: (i) shifts in dominant platforms across the observation window (e.g., growing TikTok and short-form video use), (ii) the migration of media activity to mobile devices where rule-based restriction is harder to enforce, and (iii) intensifying peer-group autonomy pressure during early-to-middle adolescence. The universality of the trajectory across heterogeneous European contexts suggests that disengagement reflects developmental processes (adolescent autonomy-seeking; Steinberg, 2001) operating regardless of cultural starting point.

The age-moderation analyses (§3.6) directly support Steinberg's (2001) developmental account: parental disengagement is not merely a period effect or methodological artifact, but reflects a structured maturational process. The strongest age effects were observed at the high-mediation end of the gradient: controlling for current profile membership, each additional year of W1 age was associated with 18%−23% lower odds of remaining in or transitioning to Active All-Round across the 2-year observation window. This is consistent with the developmental literature on parent-adolescent autonomy renegotiation (Steinberg, 2001): parents of older adolescents face increasing pressure (and adolescent expectation) to withdraw active engagement, while parents of younger adolescents retain longer-term engagement options. The Moderate Balanced profile, by contrast, showed no additional age moderation beyond what current class explained—suggesting that transitions involving Moderate may be governed by other mechanisms (e.g., parenting-style stability, family resource constraints) rather than age *per se*. Universal drift across all six countries (§3.7) combined with within-individual age effects (§3.6) jointly establish that the trajectory we observe is developmental in nature rather than contextual or methodological.

Cohen's κ values for wave-to-wave transitions (κ = 0.599 for W1 → W2 and.607 for W2 → W3, both moderate-to-substantial per Landis and Koch, 1977) indicate that mediation profiles are meaningfully stable over 1-year intervals while accommodating substantive transitions across 2 years (κ_W1 → W3 = 0.364). Profiles should thus be understood as dynamic states rather than fixed typologies, shaped by the ongoing developmental renegotiation of parent-adolescent autonomy (Steinberg, 2001).

### 4.5 Implications for theory

This study contributes to parental mediation theory in three ways. First, the finding that mediation strategies co-occur in a gradient of overall involvement—rather than in qualitatively distinct configurations such as “high-restriction/low-enabling”—suggests that the concept of “mediation intensity” may be as theoretically important as the concept of “mediation type.” If the critical distinction is between engaged and disengaged parents rather than between restrictive and enabling strategies, this would redirect theoretical attention toward the determinants of overall parental engagement (e.g., digital self-efficacy, time constraints, parenting stress) rather than the relative merits of specific strategies.

Second, the cross-national replication of the three-profile gradient across six culturally diverse countries provides evidence for structural universality: the same basic typology emerges regardless of cultural context. Simultaneously, the substantial variation in profile prevalence demonstrates cultural specificity in how these universal structures are distributed. This universal-structure/culture-specific-distribution pattern mirrors findings in general parenting research, where authoritative, authoritarian, permissive, and uninvolved parenting styles have been identified globally but with markedly different prevalence rates across cultures.

Third, the longitudinal findings introduce a temporal dimension to mediation theory. Mediation profiles are not fixed typologies but dynamic states that respond to the developmental trajectory of the parent–child relationship. The identification of disengagement as an “absorbing state” suggests that mediation theory should incorporate irreversibility—once parental digital involvement declines below a threshold, natural recovery is unlikely without external intervention.

### 4.6 Implications for practice and policy

Several practical implications emerge from our findings. First, the superiority of comprehensive mediation (Active All-Round) supports evidence-based digital parenting guidelines that encourage parents to combine clear rules with open communication, rather than relying on either strategy alone. Parenting programs could present the three profiles as a self-reflective tool, helping parents identify their current mediation approach and set goals for more balanced engagement. However, the practical readiness of a three-profile typology from a single dataset should not be overstated; replication and clinical validation are needed before such tools can be deployed.

Second, the cross-national variation underscores the need for culturally tailored interventions. Programs designed for Estonian families, where disengagement is normative (58.4%), may need to focus on motivating initial engagement, while programs for Portuguese families, where active mediation is already culturally aligned (33.6% Active All-Round), may focus on sustaining and refining existing practices. One-size-fits-all approaches to digital parenting education are unlikely to be effective across European contexts (Tan et al., 2025).

Third, the universal drift toward Disengagement across all six countries — regardless of cultural starting point—highlights the critical importance of timing. The absorbing nature of the Disengaged state (0.830–0.965 one-wave stability in every country) means that interventions to promote active digital parenting should target families early in adolescence—ideally at ages 10–13, before the absorbing trajectory takes hold. This timing recommendation applies equally to Nordic, Baltic, Central European, and Mediterranean contexts; the magnitude of drift varies, but the direction does not.

Fourth, these findings are relevant to the European Commission's Better Internet for Kids (BIK+) strategy and national digital education policies. The evidence that comprehensive mediation works best, combined with the substantial cross-national variation in profile prevalence, suggests that EU-level policies should set broad principles (e.g., promote combined rule-setting and communication) while allowing member states to adapt implementation to local cultural and digital contexts. National governments in countries with high disengagement rates (e.g., Estonia) might consider targeted awareness campaigns or incentivized parental digital literacy programs.

### 4.7 Limitations and future directions

Several limitations warrant consideration. First, all mediation measures were adolescent self-report, which may introduce perception biases, particularly among younger respondents. Parent–child dyadic data, as used by Li et al. (2024), would provide a more complete picture of mediation dynamics and allow testing of parent–child discrepancies in mediation perceptions.

Second, the ySKILLS dataset measured only restrictive and enabling mediation. A broader measurement approach—such as the six-dimensional instrument used by Sevilla-Fernández et al. (2026)—could reveal additional profiles, including technology-focused or monitoring-dominant types, that our two-dimensional measure could not capture. Replication with the EU Kids Online 2020 dataset (19 countries, *N* = 25,101, four mediation dimensions) represents a natural extension.

Third, the entropy (0.654) fell below the conventional 0.80 threshold, suggesting non-trivial classification uncertainty. While the strong external validation (η^2^ = 0.252) and the convergence with prior LPA studies provide compensating evidence, readers should interpret profile-based comparisons with appropriate caution. Future studies using Mplus's BCH method, which accounts for classification error in outcome analyses, would provide more precise estimates.

Fourth, measurement invariance across countries was not formally tested. If “restrictive mediation” carries systematically different connotations in Estonia vs. Portugal (e.g., reflecting different baseline expectations for parental involvement), then cross-national prevalence comparisons should be interpreted cautiously. Multigroup confirmatory factor analysis across the six countries is needed to establish measurement equivalence.

Fifth, data were collected during 2021–2023, overlapping with the COVID-19 pandemic, which dramatically increased children's screen time and may have temporarily altered parental mediation patterns. The longitudinal trend toward disengagement may partly reflect pandemic-related parental fatigue rather than purely developmental processes. Sixth, cross-wave attrition was substantial (42.0% W1 → W3 retention), and non-random dropout could bias longitudinal estimates.

Methodological note: All analyses were conducted in Mplus 8.3 (Muthén and Muthén, 1998–2017), including the exploratory factor analysis, single-wave and three-wave LPAs, BCH method for distal outcome analyses, and multi-group LTA with country as a known class variable. The use of Mplus FIML enabled inclusion of all participants under the MAR assumption, mitigating the differential-attrition bias that affects listwise analyses of three-wave subsamples (Enders, 2010).

Future research should extend this work in several directions: (a) applying LPA to the EU Kids Online 2020 dataset for broader cross-national coverage with additional mediation dimensions; (b) using dyadic parent–child data to examine congruence in mediation perceptions; (c) testing whether early-adolescence interventions can prevent the drift toward parental disengagement; and (d) examining whether profile-outcome associations are moderated by country, gender, or socioeconomic status.

Two additional limitations merit explicit acknowledgment. First, retention from W1 to W3 was 42.0% (*n* = 2,385 of 5,675; W1 LPA FIML base, W3 listwise numerator). Retained participants differed slightly from those lost to attrition on Wave-1 enabling mediation (*M* = 2.83 vs. 2.68, *d* = 0.15, *p* < 0.001), Wave-1 restrictive mediation (*d* = 0.09, *p* = 0.002), Wave-1 monitoring (*d* = 0.06, *p* = 0.045), age (*d* = −0.07, *p* = 0.008), and gender composition (*d* = 0.08, *p* = 0.006). Profile distribution at W1 also differed between retained and lost cases, χ^2^(2) = 13.86, *p* < 0.001: the Active All-Round share was 24.6% among retained vs. 22.3% among lost, the Moderate Balanced share was 36.8% vs. 34.3%, and the Disengaged share was 38.5% vs. 43.4%. Disengaged adolescents were thus modestly over-represented among those lost to follow-up, while Active and Moderate profiles were slightly over-represented among those retained. The longitudinal results may therefore slightly under-represent disengaged trajectories and modestly over-represent more highly mediated adolescents. The country-stratified retention rates (Estonia 45.8%, Finland 47.6%, Germany 42.6%, Italy 37.0%, Poland 23.7%, Portugal 55.7%) further imply that country-specific transition estimates carry differential precision, with the Polish estimates resting on the smallest retained subsample. Of particular note, Italy and Poland exhibited disproportionate W2 → W3 attrition (Italy: 69.0% retention at W2 dropping to 38.8% at W3, a 30 pp loss; Poland: 47.8% at W2 dropping to 26.6% at W3, a 21 pp loss); cross-national transition estimates for these two contexts should therefore be interpreted with particular caution. Second, all measurements derive from adolescents' self-reports; the construct of interest is therefore PERCEIVED parental mediation, and dyadic studies that pair adolescent report with concurrent parent report—including parent-side measures of digital competence, need for control, and parenting stress (Helsper et al., 2024) — are needed to triangulate these findings.

## 5 Conclusion

This study provides the first cross-national, longitudinal LPA-LTA evidence for a three-profile typology of parental digital mediation in Europe, established through Mplus 8.3 FIML estimation with measurement-invariant LTA, BCH-corrected distal outcome analyses, and formal multi-group testing of country moderation. The Disengaged–Moderate Balanced–Active All-Round gradient emerged robustly across six culturally diverse countries; profile prevalence varied significantly across the six countries with a small-to-medium effect (Cramér's *V* = 0.179); the Active All-Round profile predicted more favorable adolescent outcomes across six wellbeing domains via BCH analyses, though pairwise contrasts revealed a threshold rather than gradient pattern (the Disengaged–Active contrast was robust across all outcomes; Moderate–Active contrasts were largely non-significant); and longitudinal trajectories showed universal drift toward Disengagement during early-to-middle adolescence, with 2-year net Disengaged increases ranging from +12.1 pp in Portugal to +25.2 pp in Italy across the six countries. The absorbing nature of the Disengaged state across every country (one-wave stability 0.830–0.965) underscores the need for universal early-adolescence interventions to prevent disengagement before it consolidates.

## Data Availability

Publicly available datasets were analyzed in this study. The ySKILLS three-wave survey dataset can be accessed at https://data.mendeley.com/datasets/c66jczxfjc/4 (Machackova et al., 2024).
